# Does Electrophysiological Maturation Shape Language Acquisition?

**DOI:** 10.1177/17456916231151584

**Published:** 2023-02-08

**Authors:** Katharina H. Menn, Claudia Männel, Lars Meyer

**Affiliations:** 1 Research Group Language Cycles, Max Planck Institute for Human Cognitive and Brain Sciences, Leipzig, Germany; 2 Department of Neuropsychology, Max Planck Institute for Human Cognitive and Brain Sciences, Leipzig, Germany; 3 International Max Planck Research School on Neuroscience of Communication: Function, Structure, and Plasticity, Leipzig, Germany; 4 Department of Audiology and Phoniatrics, Charité – Universitätsmedizin Berlin, Berlin, Germany; 5 Clinic for Phoniatrics and Pedaudiology, University Hospital Münster, Münster, Germany

**Keywords:** oscillations, infant-directed speech, neural development, temporal speech patterns

## Abstract

Infants master temporal patterns of their native language at a developmental trajectory from slow to fast: Shortly after birth, they recognize the slow acoustic modulations specific to their native language before tuning into faster language-specific patterns between 6 and 12 months of age. We propose here that this trajectory is constrained by neuronal maturation—in particular, the gradual emergence of high-frequency neural oscillations in the infant electroencephalogram. Infants’ initial focus on slow prosodic modulations is consistent with the prenatal availability of slow electrophysiological activity (i.e., theta- and delta-band oscillations). Our proposal is consistent with the temporal patterns of infant-directed speech, which initially amplifies slow modulations, approaching the faster modulation range of adult-directed speech only as infants’ language has advanced sufficiently. Moreover, our proposal agrees with evidence from premature infants showing maturational age is a stronger predictor of language development than ex utero exposure to speech, indicating that premature infants cannot exploit their earlier availability of speech because of electrophysiological constraints. In sum, we provide a new perspective on language acquisition emphasizing neuronal development as a critical driving force of infants’ language development.

Before birth and in the first months afterward, the electrophysiological spectrum of the brain is dominated by slow activity (Fig. 1a). Delta-band activity emerges from around 24 weeks gestational age as discontinuous activity at a frequency of approximately 0.5 Hz (Vecchierini et al., 2007). The delta activity arises from the early maturation of thalamocortical connections (Arichi et al., 2017; Kidokoro, 2021). From around 31 weeks gestational age, delta activity can be exogenously evoked by sound (Chipaux et al., 2013). Somewhat earlier at around 28 weeks gestational age, slow-wave theta-band activity (4–8 Hz; TTA-SW) adds to delta. TTA-SW originates from areas close to the typical auditory and language-relevant areas (Moghimi et al., 2020). TTA-SW is insensitive to sensory input and has been argued to reflect the endogenous preparation of these areas (Routier et al., 2017). As we discuss in detail below, exogenous neural oscillations faster than the delta bands are virtually absent from the fetal brain and emerge only after birth (Anderson & Perone, 2018; Moghimi et al., 2020; Routier et al., 2017; Vanhatalo et al., 2002).

**Fig. 1.**
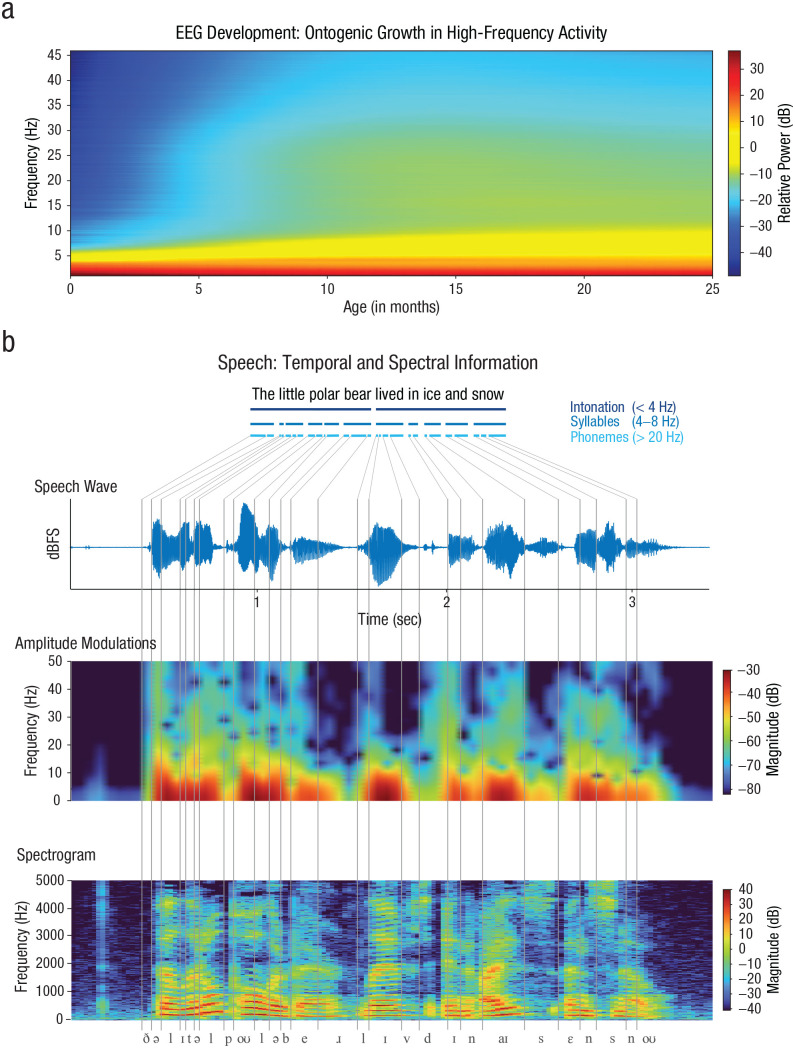
Overview of electrophysiological development across the first 2 years and the temporal and spectral information in speech. The electrophysiological activity during story listening is initially constrained to low frequencies below 10 Hz, but it speeds up across development (modelled from Menn, Männel, & Meyer, 2022) (a). Faster frequencies emerge at around 6 months. Temporal information in speech is assumed to match acoustic grain sizes of phonemes, syllables, and intonation phrases (b). The segmentation and identification of linguistic content require the modulation spectrum, obtained from the envelope, and the spectrogram, which shows spectrotemporal modulations and formant structure.

In contrast, adult speech processing is thought to use a more extended range of exogenous oscillatory activity. Exogenous oscillations in the delta and theta bands, but also faster oscillations in the gamma band (> 20 Hz), are thought to serve the temporal segmentation of speech. This may be achieved via phase alignment of the gamma, theta, and delta bands to acoustic modulations in speech (Assaneo & Poeppel, 2018; Giraud & Poeppel, 2012; Meyer, 2018; Poeppel & Assaneo, 2020). The frequencies of these bands are thought to match to the acoustic grain sizes of phonemes, syllables, and intonation phrases, respectively (Fig. 1b; Leong & Goswami, 2015; Poeppel & Assaneo, 2020). There is clear evidence that phase alignment with the corresponding acoustic modulations facilitates speech processing, as its magnitude predicts intelligibility (Peelle et al., 2013).

## The Primacy of Slowness in Early Linguistic Abilities

Newborns display selective acquaintance with slow temporal patterns characteristic of the speech prosody of their native language (Byers-Heinlein et al., 2010; Gasparini et al., 2021; Nazzi et al., 2000; Ramus, 2002). But this does not mean that newborns can yet tell their native language from any other language. Instead, they show a listening preference for speech that shares the prosodic rhythm of their mother’s language—that is, including rhythmically similar nonnative languages (Byers-Heinlein et al., 2010; Mehler et al., 1988; Moon et al., 1993; Nazzi & Ramus, 2003). Given newborns’ expertise in slow temporal patterns of speech prosody, they must acquire these patterns in utero. Indeed, hearing abilities emerge already toward the end of the second trimester of gestation (i.e., at approximately 25–29 weeks gestational age; Birnholz & Benacerraf, 1983), and the fetal brain processes speech and nonspeech sounds at least during the last trimester (Draganova et al., 2018; Hartkopf et al., 2016; Hykin et al., 1999; Muenssinger et al., 2013; Wakai et al., 1996). At this age, the auditory system is fully developed structurally and connected to the thalamus (Ghio et al., 2021; Khan et al., 2019; Takahashi et al., 2012).

## Infant-Directed Speech: Just Slow Enough for the Infant Brain?

**Fig. 2.**
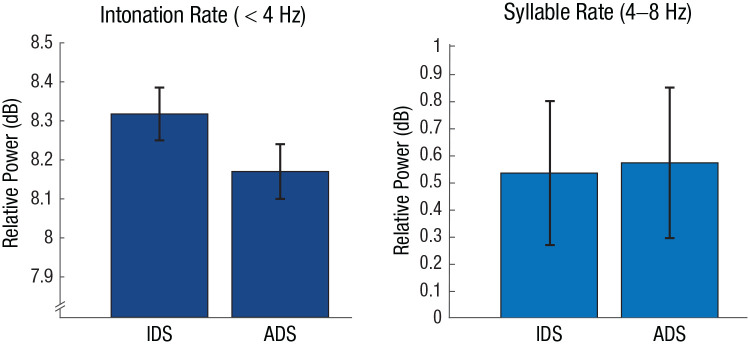
Differences in amplitude modulations between infant-directed speech (IDS) and adult-directed speech (ADS) in the intonation and syllable rate. Parents enhance amplitude modulations in the intonation rate when interacting with infants compared with interactions with adults, in line with earlier findings (Leong et al., 2017; Menn, Michel, et al., 2022). Data were taken from the Newman-Ratner Corpus in CHILDES (Newman et al., 2016). IDS data are from eight representative interactions between mothers and their 7-month-old infant, and ADS data are from interactions between the same mothers and an experimenter. Error bars indicate standard errors.

Infant-directed speech (IDS) is marked by a slow articulation rate and lengthened, hyperarticulated vowels (Casillas et al., 2020; Cristia, 2013, 2022; Fernald et al., 1989; Soderstrom, 2007; Spinelli et al., 2017). Moreover, slow amplitude modulations (< 4 Hz) are enhanced in IDS even independently of speech rate (Fig. 2; Leong et al., 2017). These adaptations have a number of behavioral benefits, such as increasing infants’ attention toward IDS compared with adult-directed speech (ManyBabies Consortium, 2020; Werker & McLeod, 1989).

Slowness also aids language development (Golinkoff et al., 2015)—presumably by amplifying those temporal patterns that infant electrophysiology is equipped to process well. A slower initial speaking rate by caregivers seems to be beneficial for language acquisition and predicts infants’ later vocabulary (Raneri et al., 2020). Most critically, infants are sensitive to caregivers’ enhancement of prosodic information and show a stronger neural phase alignment to IDS compared with adult-directed speech specifically for the prosodic rate (Menn, Michel, et al., 2022). In other words, caregivers’ enhancement of slow temporal patterns in IDS would facilitate electrophysiological processing by the delta-dominant infant brain. The emphasis on prosodic information could further highlight the parts of the acoustic speech that signals the onset of linguistic units, and it has been shown that the tracking of prosody in infancy predicts the acquisition of vocabulary (Menn, Ward, et al., 2022). This link between prosodic and linguistic processing in infants is supported by behavioral evidence of prosodic bootstrapping—the finding that infants use prosodic information to infer linguistic units (Gervain & Werker, 2013; Gleitman & Wanner, 1982; Soderstrom et al., 2003).

## Early Low-Pass Filtering of Speech: The Womb Versus the Brain

**Fig. 3.**
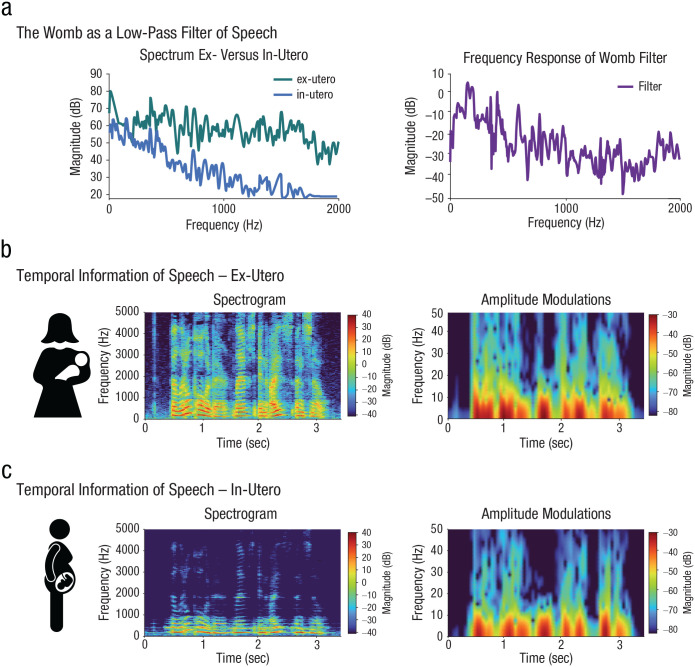
Availability of acoustic information from speech in the womb. The availability of temporal speech information inside the womb was estimated by reconstructing the womb filter from the in utero and ex utero spectrum described and depicted in Querleu et al. (1988; a). The low-pass filtering of the maternal tissue strongly affects the spectral information in speech but has less effect on amplitude modulations (b, c).

It is traditionally thought that fetuses cannot acquire temporal patterns faster than prosodic modulations mainly because the former cannot be heard in the womb (for reviews, see Gervain, 2018; Nallet & Gervain, 2021). Indeed, the maternal tissue that surrounds the fetus acts as a low-pass filter that limits the conduction of high-frequency sound (Fig. 3a). Recordings from nonhuman mammals (e.g., sheep) and results from simulation studies suggest that slow temporal patterns of speech prosody are well preserved, but frequencies above 400 to 600 Hz are strongly attenuated in the womb (Gerhardt & Abrams, 1996; Griffiths et al., 1994; Lecanuet & Granier-Deferre, 1993; Querleu et al., 1988). This suggests that the low-pass filtering of the maternal tissue strongly affects the spectral information of speech but has a limited effect on amplitude modulations, which cue onsets of new segments in continuous speech (Fig. 3b and 3c). If the acoustic filter of the womb is the only constraint on learning, fetuses should be able to exploit their prenatal exposure to segmentation cues. But this is not the case: Although newborns show phonemic perceptual learning for individually presented vowels (Wu et al., 2022), they do not yet show the ability to segment fast speech sounds from continuous speech (Bijeljac-Babic et al., 1993), indicating a maturational rather than environmental constraint on the processing of fast modulations in speech, as this information was already available in utero.

Additional evidence for maturational constraints on fast temporal information processing comes from premature infants. Given that prematurely born infants are exposed to unfiltered speech earlier than full-term infants, they should have a head start in the acquisition of faster temporal patterns, such as those required to segment phonemes. But this is not the case: Infants who are born prematurely do not tune into fast temporal patterns of their native language earlier than infants born full term—that is, they do not build native phoneme categories any earlier (Peña et al., 2012). This suggests that maturational age rather than exposure limits the time scale of processing to slow temporal patterns. This is compatible with our proposal that early language development is constrained by electrophysiological maturation: Slow oscillatory frequencies are present already in the fetal brain, whereas faster frequencies emerge only after birth, that is, after the maturation of the underlying neural tissue.

## Fast Electrophysiological Activity Emerges Late

Even after birth, slow electrophysiological activity continues to dominate infants’ electrophysiological power spectrum. Until shortly after birth, electrophysiological activity in the gamma range cannot be detected (Le Van Quyen et al., 2006). Within the first 6 months after birth, the spectrum flattens out, and the initial strong prevalence of slow electrophysiological activity decreases as high-frequency activity increases (Fig. 1a; Schaworonkow & Voytek, 2021). This is due to the gradual emergence of fast electrophysiological activity (i.e., gamma-range activity; Le Van Quyen et al., 2006). The reason why the gamma band emerges late is the ontogenetic maturation of the underlying brain tissue. In particular, the migration of GABAergic neurons continues until 6 months postnatally (Xu et al., 2011). Gamma-band activity relies on the rapid interaction between excitatory and inhibitory interneurons (Cardin et al., 2009; Fries et al., 2007). In adults, these inhibitory interneurons are mostly GABAergic (Kravitz et al., 1963; Purpura et al., 1957). Prenatally, however, differences in fetal neurochemistry cause GABA to have an excitatory effect (Dammerman et al., 2000; Gao & Van Den Pol, 2001; Owens et al., 1996, 1999). For inhibition, the fetal brain mostly relies on giant depolarizing potentials, which are too slow to allow for the emergence of faster oscillations in the gamma-range rhythms (Ben-Ari, 2002; Khazipov et al., 2004; Le Van Quyen et al., 2006).

## No Gamma, No Native Phoneme Inventory

**Fig. 4.**
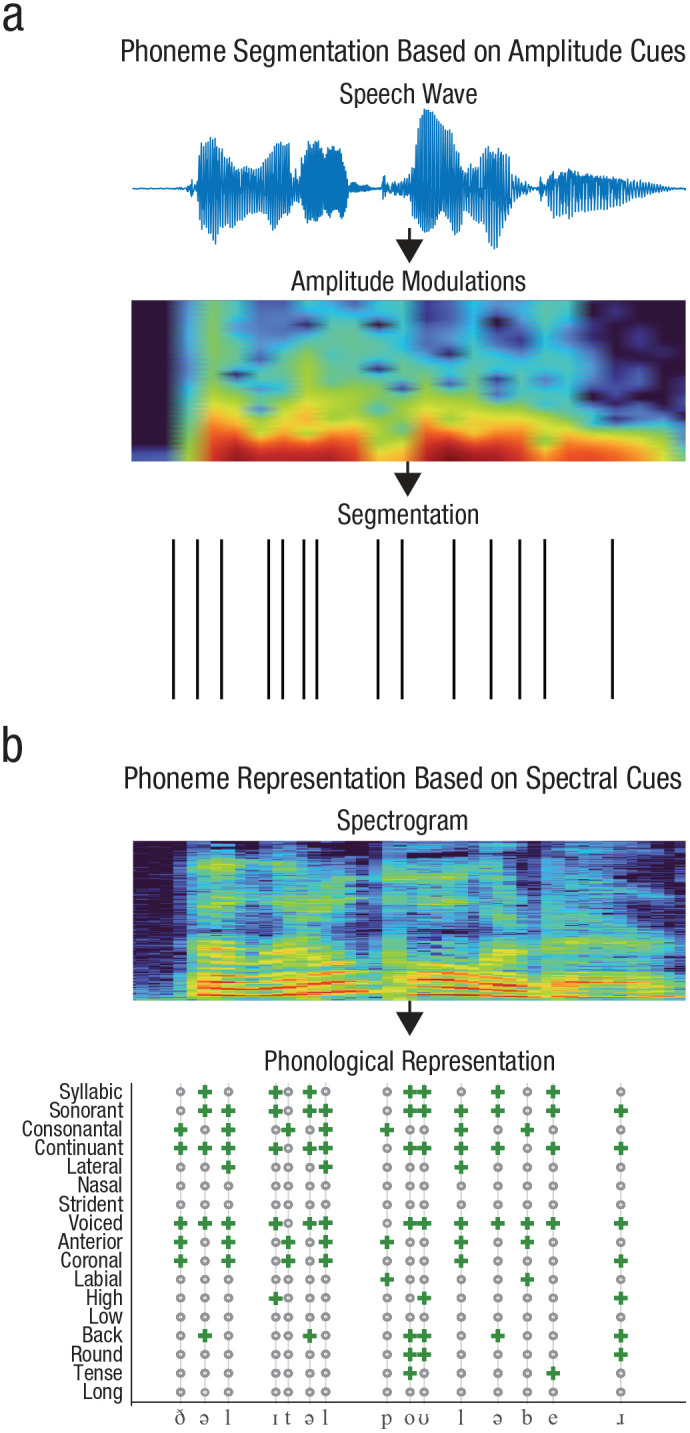
Phoneme segmentation does not equal phoneme representation. Phoneme segmentation from continuous speech is based on amplitude modulations > 20 Hz that cue phoneme onsets (a). On a neural level, segmentation is achieved by lower gamma activity tracking phoneme-rate amplitude modulations. Phonological representations are based on spectrogram information in speech (b). On a neural level, higher gamma activity is required for the representation of phonological features.

The gradual emergence of gamma-band oscillations in infancy could explain the developmental trajectory of phonological acquisition. As noted above, in adults, neural activity in the lower gamma band has been linked to the segmentation of phonemes from speech. This is thought to be achieved by phase alignment with phoneme-rate amplitude modulations (Fig. 4a; Di Liberto et al., 2015; Goswami, 2019; Gross et al., 2013).

Electrophysiological activity that could phase-align to phoneme-rate amplitude modulations emerges until the age of 6 months (Le Van Quyen et al., 2006; Xu et al., 2011). During this age precisely, infants start building an inventory of native phonemes (i.e., speech sounds; Kuhl, 2004; Tsuji & Cristia, 2014). This is indicated by the emergence of the so-called perceptual magnet effect: Infants misperceive nonnative phonemes as instances of native phonemes—that is, their native categorical knowledge exerts a top-down influence over their auditory perception. Before this age, infants can dissociate nonnative and native phonemes with equal acuity (Werker, 1995).

Before acquiring their native phonological inventory, infants must first be able to segment the acoustic segments corresponding to individual phonemes in speech. Critically, this requires a higher temporal resolution than available without the electrophysiological gamma band: Studies on adults suggest that two phonemes can be perceived only as separate acoustic segments if they are separated by at least 20 ms, such that they can trigger phase-locking of two independent gamma cycles (Giraud, 2020; Joliot et al., 1994). Strikingly, 4-day-old newborns do not discriminate bisyllabic utterances that differ only in the number of phonemes within the syllable (Bijeljac-Babic et al., 1993). Given that phoneme-rate amplitude modulations are available to the fetus in utero, this null finding indicates maturational constraints on phoneme segmentation. Even at 7.5 months of age, infants can dissociate both pure tones and phonemes only when these are at least approximately 75 ms apart (Benasich & Tallal, 2002; Partanen et al., 2013). In the visual domain, this processing slowness has even more drastic effects: 5-month-olds require an interval of more than a second to perceive two stimuli as independent (Hochmann & Kouider, 2022; Tsurumi et al., 2021). These modality-dependent effects may be an additional challenge for the acquisition of native phonemes, for which infants also rely on visual cues (e.g., mouth movements; Altvater-Mackensen et al., 2016; Burnham & Dodd, 2004; Ter Schure et al., 2016). Until now, the developmental trajectory of phoneme-rate amplitude tracking during infancy has not been assessed, but we hypothesize that neural tracking of fast temporal information develops only during infancy—and that it arises only after infants’ temporal integration windows have decreased sufficiently to perceive individual phonemes separately.

## Gamma-Band Maturation: A Critical Period for Phoneme Acquisition?

Clinical work suggests that phoneme-level acquisition and processing depends on the emergence of gamma-band activity around the age of 6 months. Six-month-old infants at familial risk for language impairments display decreased gamma-band activity during rapid auditory processing (Cantiani et al., 2019). Moreover, developmental language disorder has been related to reduced gamma-rate activity both during rest (Benasich et al., 2008) and during rapid auditory processing (Heim et al., 2013). Developmental language disorder is marked by phoneme-level difficulties that are linked to a generalized deficit in the processing of fast acoustic transitions (Corriveau et al., 2007; Richards & Goswami, 2015; Tallal & Piercy, 1973). In line with this, such difficulties are most pronounced at high speech rates, for which processing relies on even higher frequencies (Guiraud et al., 2018).

## From Low to High Gamma—From Segmentation to Representation?

So far, we have considered the role of the gamma band in phoneme-rate speech segmentation. Importantly, this role can be served only by the lower gamma band (i.e., > 25 Hz), which covers frequencies that can phase-lock to phoneme-rate amplitude modulations in speech (Leong & Goswami, 2015; O’Shaughnessy, 1995). In contrast to segmentation, the establishment of a native phoneme inventory cannot rely on the lower gamma band alone.

In adults, activity in the higher gamma band (i.e., > 70 Hz) is sensitive to phonological features (Fig. 4b; Nourski et al., 2015; Steinschneider et al., 2011). Feature processing has been related to neuronal spiking in the auditory association cortex that is sensitive to individual phonological features (Mesgarani et al., 2014).

The different proposed functional roles of the lower and higher gamma bands would entail that infants first learn to segment phonemes (= lower gamma) and afterward acquire their categorical properties (= higher gamma, spiking activity). There is indirect evidence for this idea: First, the developmental shift from slow to fast electrophysiological activity does not stop at the lower gamma band but continues until the higher gamma band (Cellier et al., 2021; Pivik et al., 2019; Schaworonkow & Voytek, 2021). Second, Ortiz-Mantilla et al. (2013) tested 6-month-old infants on their perception of native and nonnative phonemic contrasts. At this age, activity in the lower gamma band (i.e., 30–34 Hz) increased for the native compared with the nonnative contrast. At 12 months of age, this effect shifted to the higher gamma band (i.e., 70–77 Hz; Ortiz-Mantilla et al., 2016). Third, there is evidence from investigations of infants’ ability to discriminate their native language from languages with a similar rhythm. We noted above that the ability to discriminate between rhythmically similar languages (e.g., Catalan and Spanish) emerges only between 4 and 5 months of age (Bosch & Sebastián-Gallés, 1997). Because prosodic information is insufficient for the discrimination of such rhythmically similar languages, their discrimination requires sensitivity to faster, phoneme-rate modulations. Infants’ progression from a general perception of global rhythmic features to more fine-grained perception of native sound organization is accompanied by neural activity in the gamma range. Activity in the lower gamma band is seen when infants are listening to languages rhythmically similar to their native language by 3 months of age (Nacar Garcia et al., 2018; Peña et al., 2010). However, activity in the higher gamma band (55–75 Hz) increases for the native language from 6 months of age (Peña et al., 2010).

## IDS Accelerates: In Pursuit of Electrophysiological Maturation?

Although IDS is initially slow, caregivers accelerate their speech patterns across development; speech rate becomes comparable to adult-directed speech at around 2 years of infant age (Kondaurova et al., 2013; Lee et al., 2014; Narayan & McDermott, 2016; Raneri et al., 2020). In addition to adaptations of speech rate, caregivers have been found to prolong vowels in IDS (Englund & Behne, 2006; Hilton et al., 2022; Kondaurova & Bergeson, 2011; Lovcevic et al., 2020). The prolongation of vowels may ease phoneme-rate segmentation for the slow infant brain; moreover, it may help to make phonological contrasts more salient in time by slowing down the rate of phonological feature changes. Caregivers were also shown to decrease vowel duration differences between IDS and adult-directed speech as infants get older (Englund & Behne, 2006; Hartman et al., 2017; Vosoughi & Roy, 2012), possibly aligning the phoneme rate of their speech to infants’ increasing electrophysiological processing speed.

## Conclusion

We presented a new perspective on the relationship between electrophysiological maturation and language acquisition. Specifically, the prenatal prevalence of slow electrophysiological activity allows for the early development of native-specific speech processing of prosodic information, which is available to the fetus in utero. Faster electrophysiological activity in the gamma range, which is required for the segmentation and representation of native phonological information, emerges only postnatally and continues to develop across the first year after birth, therefore constraining the onset of the acquisition of acoustic-phonological knowledge to the second half of the infants’ first year. Parental speech adaptations fit the temporal granularity of infants’ electrophysiological tool kit. This provides a novel perspective for the neuroscientific investigation of language acquisition. Researchers should consider infants’ learning in relationship to their electrophysiological processing abilities as well as the speech they receive from their caregivers. Novel studies on infant’s electrophysiological speech processing should specifically focus on electrophysiological processing of frequency modulations, which are highly relevant for language development, as well as more directly on the role of gamma activity for infants’ early phonological acquisition.

## Transparency

*Action Editor:* Tina M. Lowrey

*Editor:* Klaus Fiedler
